# Scarless excision of an insertion sequence restores capsule production and virulence in *Acinetobacter baumannii*

**DOI:** 10.1038/s41396-021-01179-3

**Published:** 2021-12-23

**Authors:** Clémence Whiteway, Adam Valcek, Chantal Philippe, Mojca Strazisar, Tim De Pooter, Ivan Mateus, Anke Breine, Charles Van der Henst

**Affiliations:** 1 Microbial Resistance and Drug Discovery, VIB-VUB Center for Structural Biology, VIB, Flanders Institute for Biotechnology, Brussels, Belgium; 2 Structural Biology Brussels, Vrije Universiteit Brussel (VUB), Brussels, Belgium; 3 Research Unit in the Biology of Microorganisms (URBM), NARILIS, University of Namur (UNamur), Namur, Belgium; 4 Neuromics Support Facility, VIB Center for Molecular Neurology, VIB, Antwerp, Belgium; 5 Department of Biomedical Sciences, University of Antwerp, Antwerp, Belgium; 6 Department of Ecology and Evolution, University of Lausanne, Lausanne, Switzerland

**Keywords:** Bacterial genetics, Bacterial pathogenesis

## Abstract

We identify a new mechanism mediating capsule production and virulence in the WHO and CDC priority ESKAPE pathogen *Acinetobacter baumannii*. Non-capsulated and avirulent bacteria can revert into a capsulated and virulent state upon scarless excision of an IS*Aba13* insertion sequence under stress conditions. Reversion events fully restore capsule production and in vivo virulence. This increases our knowledge about *A. baumannii* genome dynamics, and the regulation of capsule production, virulence and resistance.

## Introduction

*Acinetobacter baumannii* is an opportunistic human pathogen and a constant growing threat because of its propensity to aquire multidrug resistance [1]. For this ESKAPE pathogen [2], ranked as critical priority by WHO [3] and CDC [4], new antimicrobial strategies for prophylaxis and treatment are urgently needed. Despite its clinical relevance, only little is known about *A. baumannii* overall virulence and its regulation [5, 6]. However, production of envelope determinants including the exopolysaccharide capsule is critical to escape the host immune system, to resist desiccation and antimicrobial treatments [6]. Genes required for biosynthesis and export of exopolysaccharides are clustered within the capsule locus (K-locus). Capsule composition and structure are highly variable between *A. baumannii* isolates. So far, at least 128 different K-locus types [7] have been identified and over 40 K units structure have been elucidated [8]. In addition to the high genetic diversity amongst isolates, phenotypic heterogeneity is generated in clonal populations by a high frequency phenotypic phase variation mechanism, impacting capsule production in *A. baumannii* which allows interconversion between virulent (VIR-O) and avirulent bacteria (AV-T) [9–12].

Here, we identify a new mechanism that controls the virulence and resistance of *A. baumannii*, which modulates capsule formation by the insertion/excision of the insertion sequence (IS) element IS*Aba13*. Our study therefore contributes to an increased knowledge about genome plasticity and virulence and resistance regulation in *A. baumannii*.

**Fig. 1:**
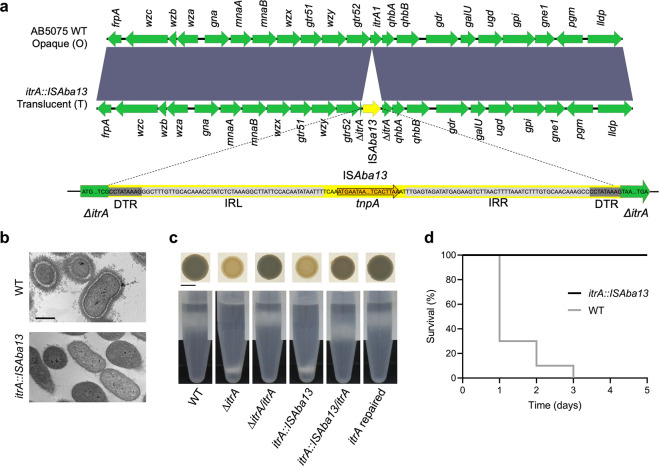
Comparison of the WT and *itrA::ISAba13* strains. **a** Sequence alignment of the K-loci of the WT and *itrA::ISAba13* mutant. IS*Aba13* (represented in yellow) interupts *itrA* gene. IRR/IRL: respectively, inverted repeat right and left, *tnpA*: transposase gene, DTR: Direct Target Repeats of 9 nucleotides flanking IS*Aba13*. Dark gray color indicates 100% sequence identity. **b** TEM of the WT and *itrA::ISAba13* strains. Scale bar: 500 nm. **c** Macrocolonies and density gradients. Scale bar: 0.5 cm. **d** Survival of *G. mellonella*. *Y* axis: survival of larvae (%), *x* axis: time post-inoculation (days), 10 larvae were infected for each condition and experiments were performed in biological duplicates.

We characterized at the genetic and phenotypic levels two *Acinetobacter baumannii* isolates derived from the modern and broadly used parental AB5075 reference strain [13], received from two different laboratories, which exhibited different behaviors on solid media. For the first one, renamed AB5075-VUB (WT), we mostly observe opaque and mucoid colonies with occasional transient translucent and non-mucoid colonies, as previously described [10]. Whereas for the second one, named AB5075-VUB-*itrA::ISAba13*, only stable translucent bacteria are observed. We sequenced and compared de novo assembled whole genomes for both strains (Supplementary Table. 1). The major difference detected within the capsule locus (KL25) is an IS*Aba13* Insertion Sequence (IS) interrupting the *itrA* gene of the *itrA::ISAba13* mutant (Fig. 1a and Supplementary Table. 2). Duplicated Target Repeats (DTR) of 9 nucleotides are found at both extremities of the IS*Aba13* element. The *itrA* gene encodes for the initial glycosyltransferase A required for both capsule assembly and O-linked protein glycosylation [14]. According to ItrA loss of function phenotypes [14], we predicted the insertion to inactivate *itrA* gene and therefore impair capsule production and virulence.

We used transmission electron microscopy (TEM) combined with capsule staining to directly visualize the exopolysaccharide capsule in the WT and *itrA::ISAba13* strains. This assay confirms the production of a thick capsule by WT cells and the absence of capsule surrounding *itrA::ISAba13* bacteria (Fig. 1b). To assess capsule formation in a fast and semi-quantitative way, we implemented a density gradient based method for *A. baumannii* bacteria [15, 16]. This density gradient confirms the phenotypes observed in TEM (Fig. 1c and Supplementary Fig. 1). We generated the deletion and complementation strains that validate the phenotype observed and its direct link to the disruption of the *itrA* coding sequence. The *itrA* gene was cloned at the neutral *attTn7* site under the control of a constitutive promoter [17] (Fig. 1c and Supplementary Table. 1). Opacity of colonies correlates with production (opaque phenotype) and lack (translucent phenotype) of capsule (Fig. 1c). The WT and *itrA::ISAba13* mutant have similar growth in liquid medium (Supplementary Fig. 2a, b), even though we systematically observe a significantly reduced area for the macrocolonies of the *itrA::ISAba13* strain compared to the WT strain on solid media (Supplementary Fig. 3).

We next assessed the in vivo virulence of the different strains using *Galleria mellonella* larvae [18]. We confirm the WT as fully virulent, all the larvae being killed after three days post-inoculation, whereas all the larvae survive with the avirulent *itrA::ISAba13* mutant after 5 days post inoculation (Fig. 1d). As controls, artificial deletion of *itrA* (∆*itrA*) in the WT strain abolishes virulence and capsule formation whereas complementation or in situ reparation of *itrA* in the *itrA::ISAba13* mutant restores the virulence phenotypes (Supplementary Fig. 4).

**Fig. 2:**
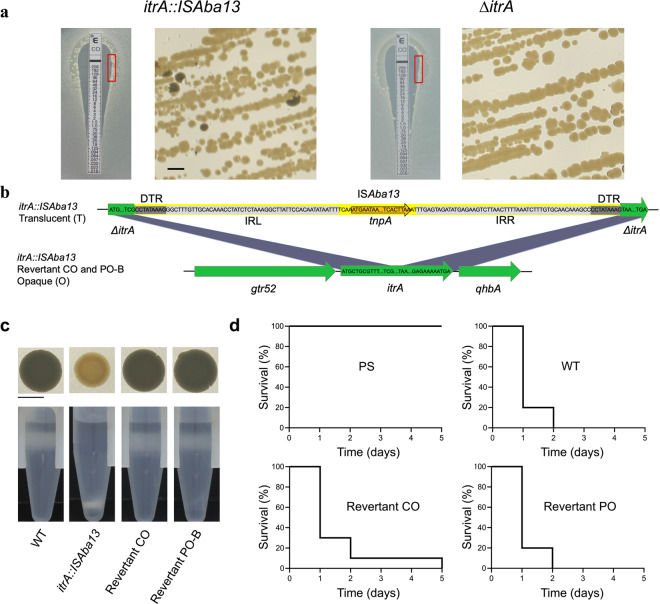
IS*Aba13* scarless excision. **a** Opaque colonies isolated by plating *itrA::ISAba13* bacteria harvested near the inhibition zone of a Colistin Etest. Bacteria near the inhibition zone (red square on left panel) were collected with a plastic loop and re-isolated on solid medium (right panel). The non-reversible control ∆*itrA* strain shows 100% of translucent colonies. Scale bar: 0.2 cm. **b** Sequence alignment of the K-loci of the *itrA::ISAba13* natural mutant strain and Revertant CO and PO-B. **c** Macrocolonies phenotype on solid media and density gradients. Scale bar: 0.5 cm. **d** Survival of *G. mellonella* over time. *Y* axis: survival of larvae (%), *x* axis: time post-inoculation (days). 10 larvae were infected for each condition and experiments were performed in biological duplicates.

The natural *itrA::ISAba13* mutant is stably deficient for capsule production and associated virulence under rich laboratory conditions. Since the lack of capsule in various stresses and infectious conditions can be deleterious for bacteria [19], we assessed the stability of the *itrA::ISAba13* phenotype. The presence of identical DTR flanking both IS*Aba13* extremities suggests that a scarless excision is possible, therefore reconstructing a WT and functional *itrA* gene copy [20]. However, for the translucent *itrA::ISAba13* strain, we do not detect any opaque colonies on solid rich media on a total of 2.8 × 10^5^ colonies monitored. In addition, the colonies remain translucent after 4 successive passages in liquid media (Supplementary Fig. 5). As the liquid cultures are done in a rich medium, optimized for bacterial growth with minimal stress exposure, we decided to assess the stability of the phenotype in more stressful conditions. We inconsistently detect very rare opaque revertant clones in bacterial lawns produced by the *itrA::ISAba13* strain after 6 days of incubation on saturated solid medium (2.5 × 10^4^ colonies counted in total) while no opaque colony are detected for the non-reversible ∆*itrA* deletion strain (5.1 × 10^4^ colonies counted in total), showing that natural reversion events can occur, but at very low frequency in the tested conditions (Supplementary Table. 3). We then used polymyxins, which are last resort antibiotics against multidrug-resistant *A. baumannii* infections [21], as additional selective pressure. Moreover, bacterial capsule is involved in the resistance to these antibiotics [22]; while in a recent study, antibiotic treatment on *A. baumannii* biofilms correlates with a strong activity of IS*Aba13* [23]. We tested E-test strips (Biomérieux) of polymyxin B and colistin deposited on *itrA::ISAba13* bacterial lawns. Under these conditions, we consistently detected opaque revertant clones after restriking bacteria surrounding the inhibition zone, with both antibiotics (Fig. 2a). Controls rule out any contamination possibility with a WT strain (Supplementary Fig. 6) and in addition, the ∆*itrA* artificial deletion strain, lacking the reversion ability, does not generate any opaque clones (Fig. 2a). PCR and sequencing of the *itrA* coding sequence on both colistin (Revertant CO) and polymyxin B (Revertant PO-B) revertants confirm the excision of the IS*Aba13* element and that the WT coding sequence is completely restored in a scarless manner (Fig. 2b and Supplementary Fig. 7). Accordingly, capsule production (Fig. 2c) and virulence (Fig. 2d) are restored to the WT level for the *itrA::ISAba13* revertants. The fate of the IS*Aba13* insertion sequence after excision remains to be determined. After showing that reversion is qualitatively possible, we determined that it is a low frequency process (Supplementary Table. 3). The precise signal(s) regulating the occurrence and the frequency of both the integration and excision events remain(s) to be identified.

The reversion mechanism described in our study differs from the previously identified transient phenotypic phase variation [12]. The stability of the non-capsulated *itrA::ISAba13* phenotype (Supplementary Fig. 5, 8, 9) can be beneficial for the bacterial population to survive infection by bacteriophages targeting the exopolysaccharide capsule of *A. baumannii*, against which non-capsulated bacteria were already shown to resist their predation [24]. Therefore, the presence of a constant pool of sensitive preys sustaining the bacteriophage population is minimized compared to a higher frequency phenotypic phase variation. However, polysaccharide capsule plays key roles in other bacterial resistances and virulence. Hence, a reversion mechanism, such as the one here identified, is required to restore capsule formation, and the associated resistance and virulence abilities. Such reversion events, fostered by stress condition such as antibiotics, can represent a broad adaptative mechanism in microorganisms. A previous study described the modulation of cell surface sialic acid in *Neisseria meningitidis* by the insertion/excision of IS1301 (from the IS5 familly) in the *siaA* gene involved in capsule expression and endogenous LOS sialylation [25]. Concerning *A. baumannii*, out of 246 complete chromosome sequences available to date in GenBank, partial or complete insertion sequence within K-loci is identified in 23 isolates (9.31%) (see Supplementary Table. 4), showing that IS elements can contribute to the locus dynamics and capsule regulation in *A. baumannii*. Moreover, IS*Aba13* IS are identified in a significant proportion (55 out of 246) of all the published genomes of *A. baumannii*, and several other IS elements are commonly present as potential key contributors to the genome dynamics [26].

Our study also highlights that every *A. baumannii* isolate exchanged deserves a careful and in-depth monitoring at the genetic level, using whole genome sequencing, along with a constant tracking of bacterial stocks.

## Methods

### Bacterial strains and growth conditions

Bacterial strains and plasmids used in this study are listed in Supplementary Table. 1. Bacterial cultures were initiated from a single clone and were grown at 37 °C in 5 ml of liquid broth low salt (LB_LS_, Luria-Bertani formulation) from Duchefa Biochemie under agitation (166 rcf) or on solid LB-agar plates (25 ml) unless indicated otherwise. 30 µg/ml of Apramycin sulfate salt (Sigma–Aldrich) or 10% of crystalized sucrose (Duchefa Biochemie) were used for the selection/counter selection of recombinant/ transformant clones. Four consecutive passages (~64 h) were done in liquid culture to assess the phenotypical stability of the *itrA::ISAba13* isolate as described in Supplementary Fig. 5. Eight hours and 16 h cultures were alternated twice and CFUs were plated from the initial bacterial culture and from the final passage. Quantification of height and area were measured using ImageJ. Statistical analyses were performed using unpaired *t* test with GraphPad Prism 9. All experiments were carried out in biological triplicates unless stated otherwise.

### Genome assembly and K-loci comparison

AB5075-VUB and *itrA::ISAba13* are two clonally isolated strains originating from the parental AB5075 reference strain [13] (Supplementary Table. 1). They were called according to the field nomenclature, renaming the sub-cultured strains by adding “-VUB” to the strain name [27]. Their complete genomes were sequenced, *de novo* assembled and compared. Genomic DNA was extracted using the QIAGEN Genomic-tip 100/G following the manufacturer’s recommendations. The short reads from MiSeq (Illumina) were trimmed for quality (Q ≤ 20) and adaptor residues using Trimmomatic V0.36 [28] (Q ≤ 20; read length ≤8; sliding window 4:20). The long reads from MinION (Oxford Nanopore Technologies) were demultiplexed and base-called using Guppy v3.2.2 (high accuracy model) and subsequently were adaptor and quality (Q ≤ 8) trimmed using Porechop v0.2.2 (https://github.com/rrwick/Porechop) and BBDuk (https://sourceforge.net/projects/bbmap/) with default settings, respectively. The short reads were used to polish the long reads employing Ratatosk v0.7.0 [29] with default settings. The corrected long reads were then de novo assembled using Flye [30] v2.9 resulting in circular chromosomal contigs. The circular chromosomal contigs were polished using long reads via racon v1.4.20 (https://github.com/isovic/racon) and Medaka v1.0.3 (https://github.com/nanoporetech/medaka) and subsequently using the short reads in two rounds of Pilon [31] v1.24 polishing. The mean coverage of short and long reads for AB5075-VUB was 41x and 340x and for AB5075-VUB-*itrA::ISAba13* the mean coverage was 37x and 490x, respectively. The K-loci of the WT and *itrA::ISAba13* were typed using Kaptive v0.7.3 (*A. baumannii* database downloaded in May, 2021), compared by pair-wise alignment in Geneious R9 (Biomatters, New Zealand) [32] and visualized in EasyFig [33] v2.2.2. The complete genomic sequences and corresponding sequencing data (including capillary sequencing data of amplicons) were deposited in GenBank under BioProject PRJNA701627. In order to verify the assemblies, the short and long reads were mapped to the region of *itrA* in AB5075-VUB (Supplementary Fig. 8) and to region of IS*Aba13* disrupting *itrA* in AB5075-VUB-*itrA::ISAba13* (Supplementary Fig. 9). The visualization was performed in Geneious R9.

### Cloning and generation of mutant strains

Plasmids and primers used to generate the strains used in this study are, respectively, listed in the Supplementary Table 1 and 5. Primers were purchased from Integrated DNA Technology (IDT) and sequencing was done using the Mix2Seq Kits – Overnight from Eurofins (Sanger sequencing). Gene deletion and complementation at the *attTn7* site of AB5075-VUB were carried out following an adapted version of the previously published protocols [17] to obtain marker-less mutants. Complementation were done at the predicted neutral *attTn7* site [34] and the genes were cloned under the influence of the artificial constitutive promoter P_strong_ (modified Ptac without lacO sites) and the RBS of the superfolder GFP (sfGFP) [17]. All constructs were introduced using natural transformation according to the previously published protocol [35]. Briefly, a large chimeric DNA fragment carrying the *sacB-aaC* selection/counter-selection cassette flanked by 1–2 kb homologous regions upstream and downstream of the targeted site was inserted at the locus of interest. The *itrA* coding sequence, the homologous regions flanking *itrA* and the att*Tn7* site were amplified by PCR from gDNA, the *sfGFP* coding sequence and P_strong_ promoter from the pASG-1 plasmid and the *sacB-aaC* cassette from the pMHL2 plasmid. PCR were performed using PrimeStarMax; TaKaRa (high fidelity DNA polymerase) and the resulting products were checked on agarose gel (1%) and purified using the Wizard SV Gel and PCR Clean-Up System (Promega) following the manufacturer’s recommendations. The final chimeric DNA fragments were generated using the NEBuilder HiFi DNA Assembly Master Mix to sew the independent fragments. First the fragment containing the *sacB-aaC* cassette was introduced at the targeted locus and the recombinant strain selected on Apramycin 30 µg/ml, was then transformed with a chimeric product containing the final desired fragment without any marker and counter selected on LB without NaCl (10 g/L tryptone, 5 g/L yeast extract) – agar 10% sucrose and incubated 6 h at 30 °C.

### Colony morphology

The colony morphology was assessed by spotting 5 µl of stationary phase bacteria from O/N culture on LB_LS_ agar plates containing a volume of 25 ml of medium. After incubation (non-inverted) at 37 °C for 24 h, back light pictures of the petri dishes were taken with a Canon hand camera. Area of the macrocolonies were analyzed using ImageJ.

### Density gradient

LUDOX Colloidal Silica (30 wt. % suspension in H_2_O, Merk) was used to semi-quantify capsule production of the different strains. The gradient solution was autoclaved before use and stored at 4 °C. 1 ml of O/N culture was centrifuged for 5 min at 5000 rcf. The pellet was suspended in 1 ml of PBS. 750 µl of PBS resuspended bacteria were mixed with 250 µl of LUDOX colloidal silica. This mix was then centrifuged for 30 min at 9000 rcf. Pictures were taken directly after the centrifugation using a Canon hand camera in front of black background.

### Transmission electron microscopy (TEM)

Bacteria were fixed and stained according to the previously published protocol [10]. After the last treatment with the 1:1 mix of oxide propylene, the fixed pellet of bacteria was transferred into a cupule to be embedded in resin polymerized 12 h at 37 °C, 48 h at 45 °C and finally three days at 60 °C. The resin containing the bacteria was then cut into ultrathin sections (~60 nm), deposited on an electron microscopy grid and stained with acetate uranyl.

### *Galleria mellonella* infections

Larvae were purchased from BioSystems Technology, TruLarv, stored at 15 °C and used within the 5 days after arrival. Bacteria were washed twice in physiological saline (PS: 0.9% NaCl in H_2_O) solution and diluted to a concentration of 1.10^7^ CFU/ml. Before injection, the larvae were incubated for 30 min at 4 °C. 10 µl of bacterial suspension in PS (1 × 10^5^ CFU/ml) were injected in the last left proleg of the larvae using a 0.3 ml insulin syringes (BD MicroFine). 10 larvae were in inoculated per replicate. One control group was injected with PS as negative control and another group with the virulent AB5075-VUB WT as positive control of virulence. Survival was monitored every day over a 5 days period by checking the keratinization and mobility phenotypes. Infection experiments were done in biological duplicates, with a total of 20 larvae tested for each condition.

### Detection of phenotypical reversion

To assess the *itrA::ISAba13* phenotypic stability, we cultivated the bacteria in a dense lawn on solid LB medium for 6 days at 37 °C. Bacterial lawns were prepared by spreading 5 ml of stationary phase bacteria from O/N culture diluted to an OD of 1 (corresponding concentration ~3 × 10^8^ bacteria/ml). After 20 s the excess of liquid was removed, and the plates were dried and incubated at 37 °C for 6 days. The *∆itrA* strain was used as a negative control of non-reversible phenotype. The *itrA::ISAba13/sacB-aaC* and *itrA::ISAba13/sfGFP* strains were used as additional controls, each of them carrying genetic markers, to show the absence of contamination by the marker-less WT strain. The presence or absence of IS*Aba13* in the *itrA* coding sequence was verified by PCR using the IS13V2_fw and IS13V2_rev primers (see Supplementary Table. 5). To assess the influence of antibiotics on the reversion events, similar bacterial lawns were prepared and Colistin Etest CO 256 WWB30 and Polymyxin B Etest purchased from Biomérieux were deposited on the dried bacterial lawn surface and incubated at 37 °C for 6 days. Bacteria were then collected from the inhibition edges and resuspended in PBS, diluted and plated on LB-agar. After 24 h of incubation at 37 °C, back light pictures of the petri dishes were taken using a Canon hand camera and colonies were screened for translucent (T) and opaque (O) phenotypes. Opaque clones were isolated and IS*Aba13* insertion in *itrA* was screened by PCR, then sequenced to verify the *itrA* coding sequence.

## Supplementary information

Supplemental Material
