# Supplementary material for: Scarless excision of an insertion sequence restores capsule production and virulence in Acinetobacter baumannii
Source: ISME J. 2021 Dec 23;16(5):1473–7. doi: 10.1038/s41396-021-01179-3 (PMC9038732; doi:10.1038/s41396-021-01179-3)
Supplement: Supplementary file 1 — Supplemental Material [file 41396_2021_1179_MOESM1_ESM.docx]

**
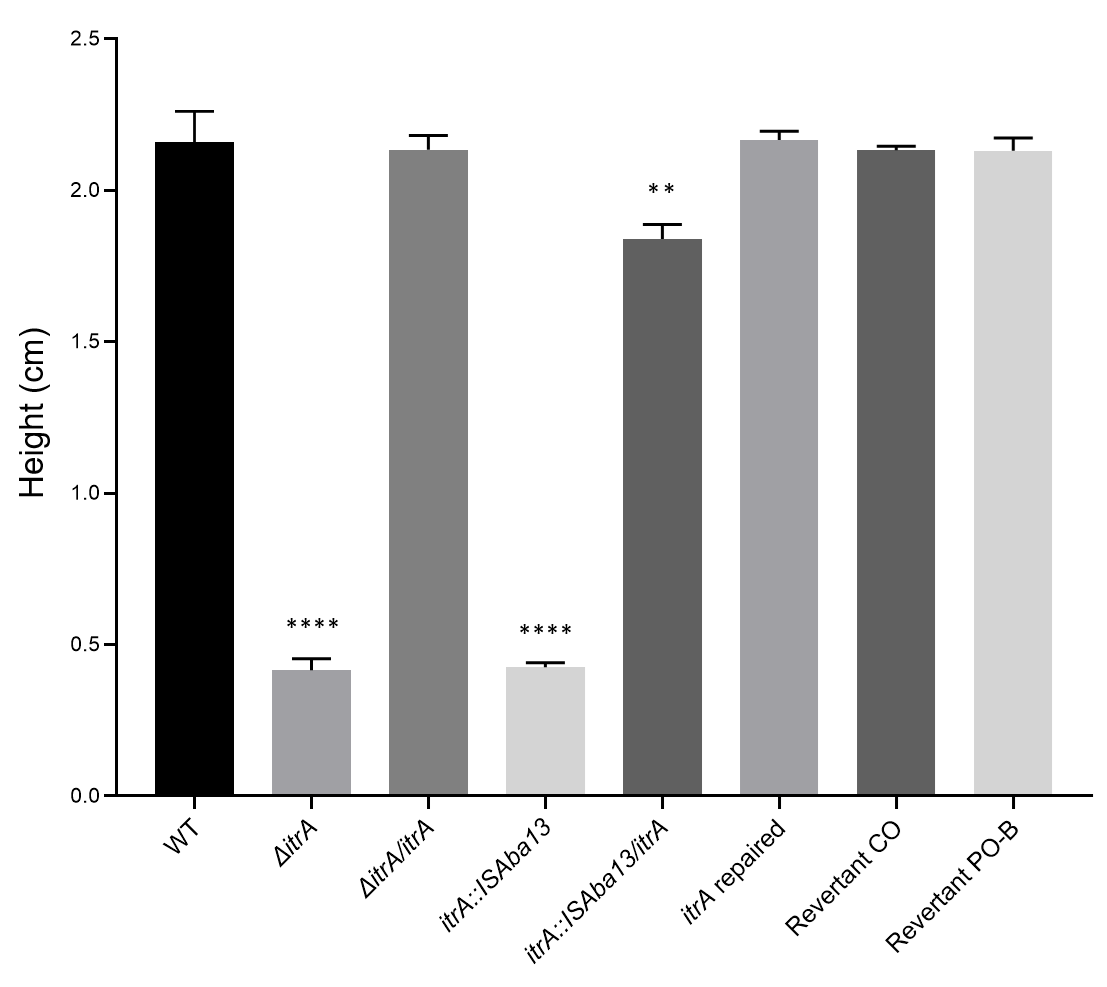
**

**Supplementary Fig.1 | Semi-quantification of capsule production using density gradients.** The height of the band (cm) was measured from the bottom of the tube (y axis) for the different strains (x axis). The different groups were compaired using a one-way ANOVA and showed a significant difference among means: (P-value < 0.0001 and F= 758.9). Then unpaired student tests (t-test) were performed on the data by comparing each strain to the WT (reference). Difference is significant if the P-value (P) <0.05. GP(GraphPad): P-value>0.0332 (*), p-value>0.0021 (**), p-value>0.0002(***), p-value<0.0001(****).

**
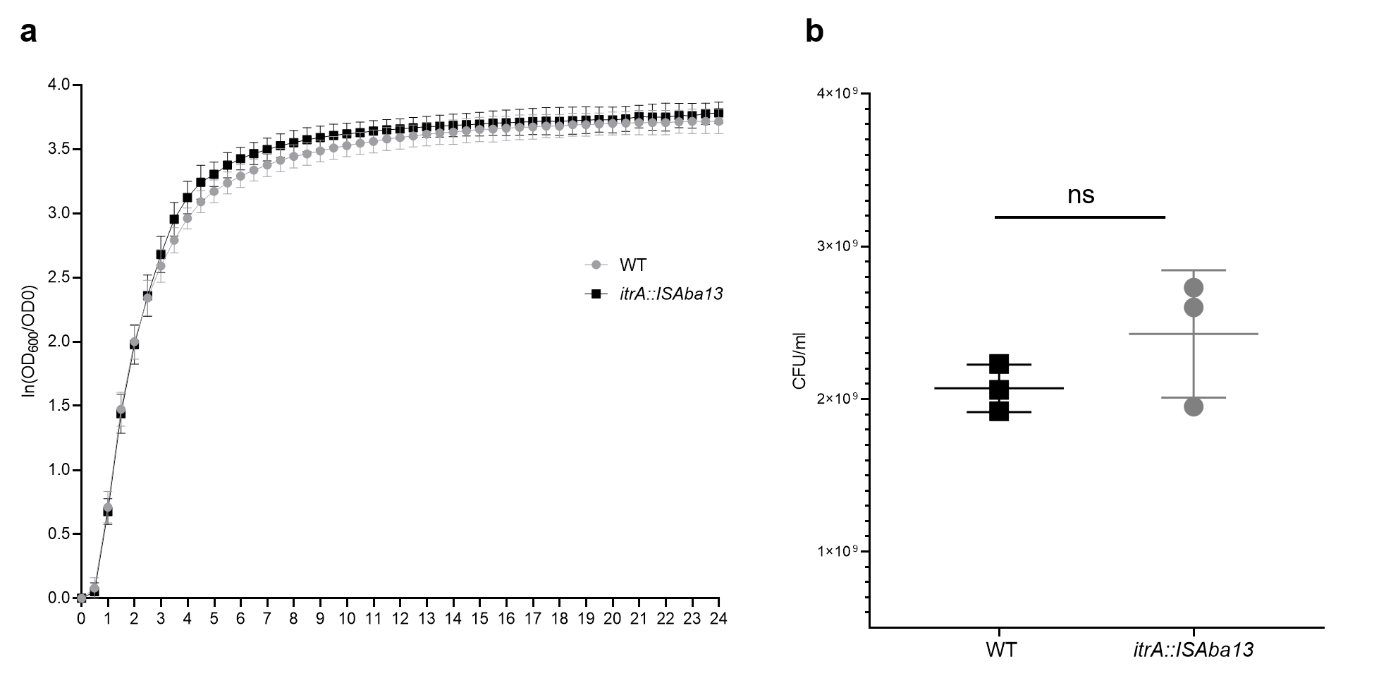
**

**Supplementary Fig.2 | Growth measurements of AB5075 WT and *itrA::ISAba13*. a**, Growth curves of AB5075 WT and *itrA::ISAba13* after 24h of measurements using a Cytation 1 (BioTek, United States). **b**, Colony forming units (CFU/ml) obtained by plating a stationary phase overnight culture of the WT and *itrA::ISAba13* (16h incubation at 37°c). An unpaired student test (t-test) was performed on the data by comparing the WT and *itrA::ISAba13* strains CFUs. No significant (ns) difference is observed between the two strains (P>0.05).

**
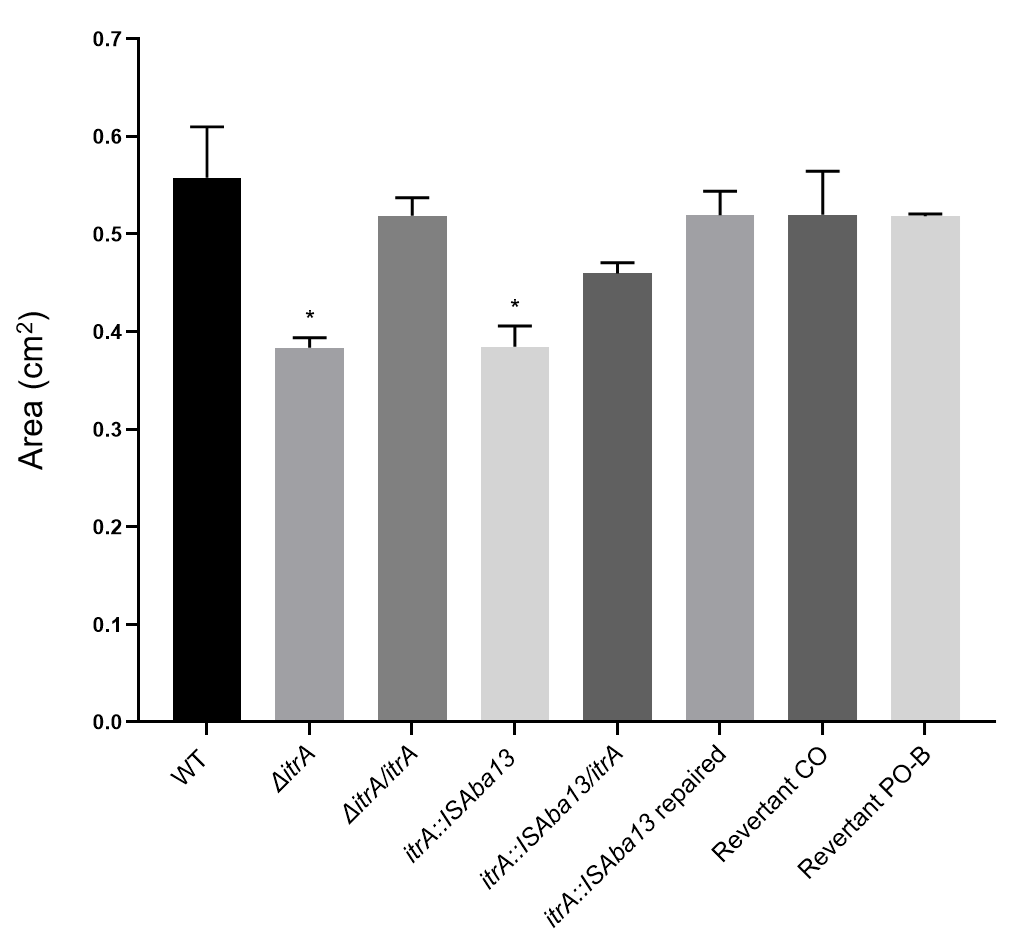
**

**Supplementary Fig.3 | Area of macrocolonies on solid media after 24h at 37°C.** The area of the macrocolonies was measured using ImageJ. The different groups were compaired using a one-way ANOVA and showed a significant difference among means: (P-value < 0.0001 and F= 16,71). Unpaired student tests (t-test) were performed on the data by comparing each strain to the WT (reference). Difference is significant if the P-value (P) <0.05. GP(GraphPad): 0.05<P-value (P) <0.0332 (*)

**
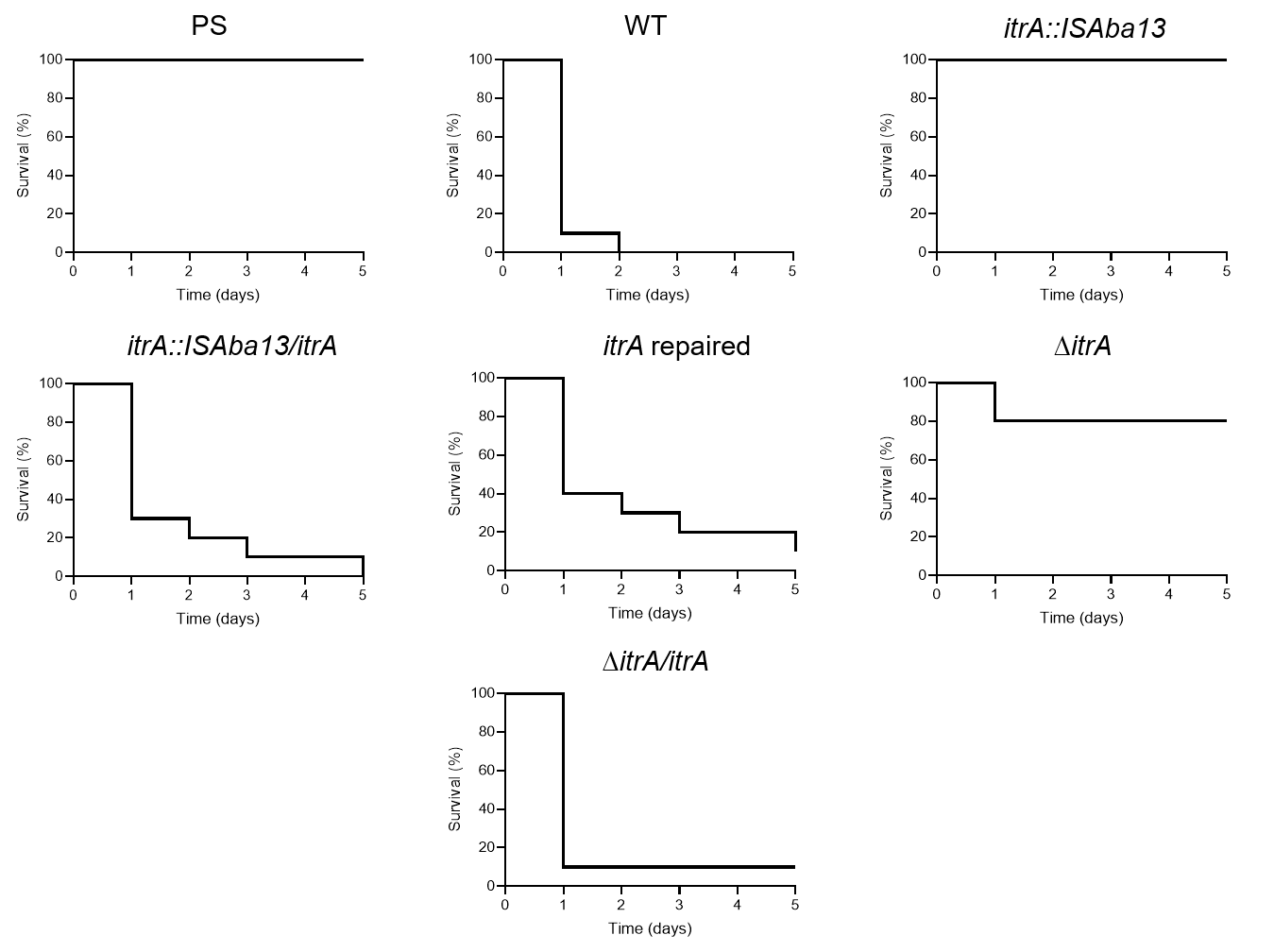
**

**Supplementary Fig.4 | Survival of *G. mellonella* moth larvae over time after inoculation with WT and *itrA::ISAba13* deletion and complementation strains**. Survival of the larvae was monitored every day for 5 days. Y-axis: survival of larvae (%), x-axis: time post-inoculation (days). 10 larvae were infected for each condition and experiments were performed in biological duplicate.

**
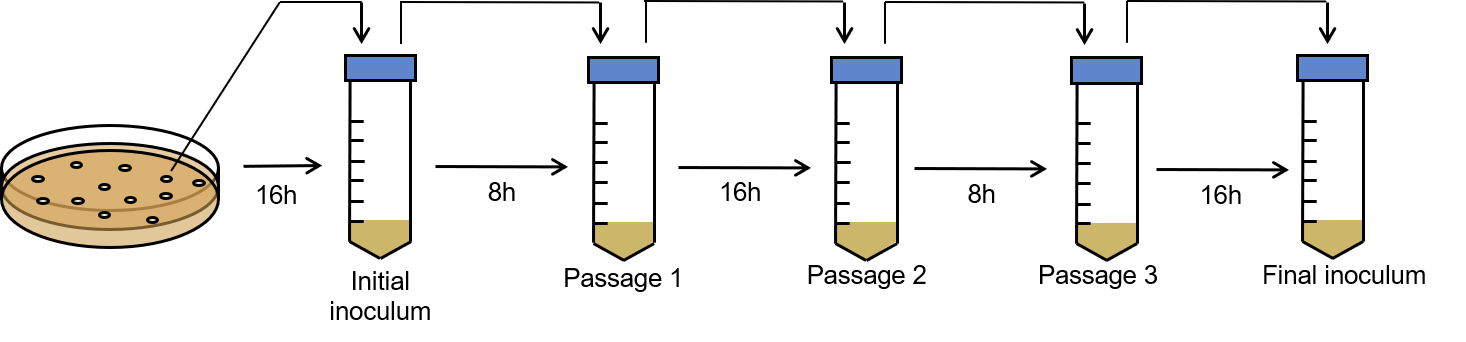
**

**Supplementary Fig.5 | Schematic representation of the successive culturing of *itrA::ISAba13.*** Four consecutive passages (~64 hours) were done in liquid culture to assess the phenotypical stability of the *itrA::ISAba13* natural mutant strain. CFUs were plated from the initial bacterial culture and from the final passage.


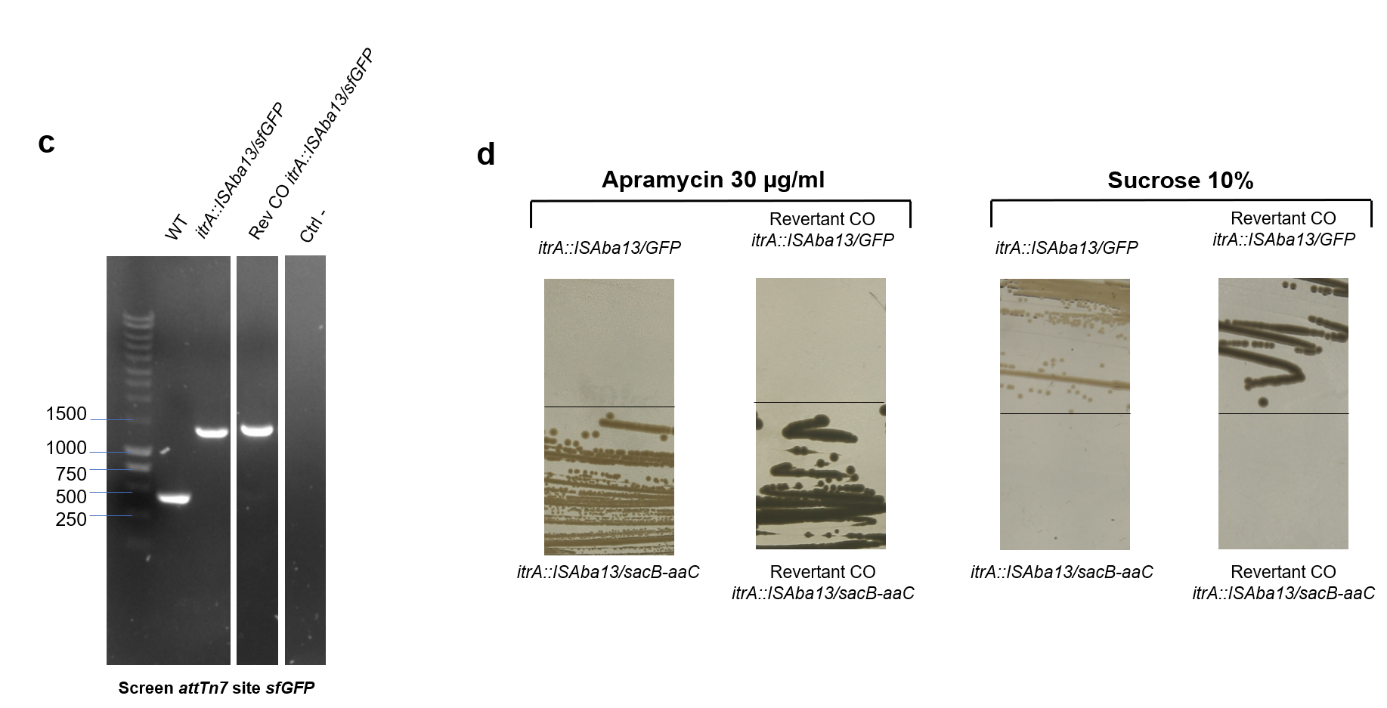


**Supplementary Fig.6 | Screening and identification of revertant clones.** Revertant clones were obtained from Colistin Etest after 6 days incubation at 37°C using the *itrA::ISAba13/sacB-aaC* and *itrA::ISAba13/sfGFP*. PCR and gel electrophoresis were done to screen **a**, for excision of IS*Aba13* (IS3V2_for and IS13V2_rev) size with IS*Aba13* inserted: 1823 bp, without insertion: 784 bp ; and to **b**, screen the Tn*7* for: presence of *sacB-aaC* with: upstream part (UP): Tn7_checkUP_for and Tn7_checkUP_rev (775 bp) and downstream part (DOWN): Tn7_checkDOWN_for and Tn7_checkDOWN_rev (575 bp); or **c**, *sfGFP* with; Tn7_checkUP_for and Tn7_checkDOWN_rev (1366 bp). **d**, Phenotypes of revertants from *itrA::ISAba13/sacB-aaC* and *itrA::ISAba13/sfGFP* on Apramycin (30µg/ml) or sucrose (10%). See Supplementary Table. 2 for primers sequences.


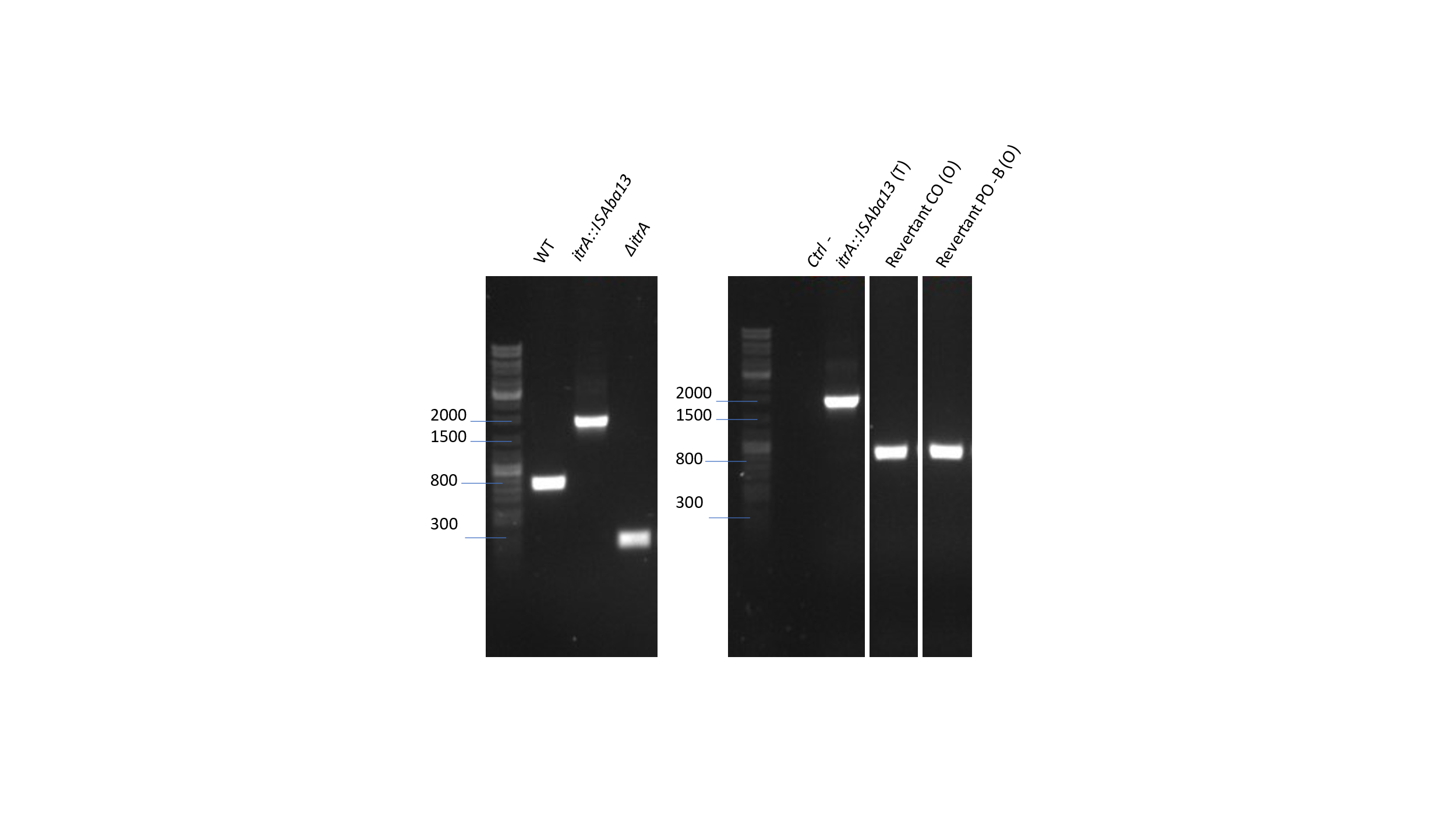


**Supplementary Fig.7 | Screening and identification of revertant clones.** Revertant clones were obtained from Colistin Etest (CO) and Polymyxin B Etest (PO-B) after a 6 days incubation at 37°C and re-isolating clones on solid medium using the *itrA::ISAba13* strain. PCR and gel electrophoresis were done to screen for excision of IS*Aba13* (IS3V2_for and IS13V2_rev), size with IS*Aba13* inserted: 1823 bp, without insertion: 784 bp, size with deletion of *itrA*: 296 bp. See Supplementary Table. 2 for primers sequence. T: translucent clone, O: opaque clone. The PCR fragment obtained with the Etest (CO) and Etest (PO-B) were Sanger sequenced using IS3V2_for and IS13V2_rev primers (Mix2Seq Kits – Overnight from Eurofins). The data were deposited in SRA within the BioProject PRJNA701627.


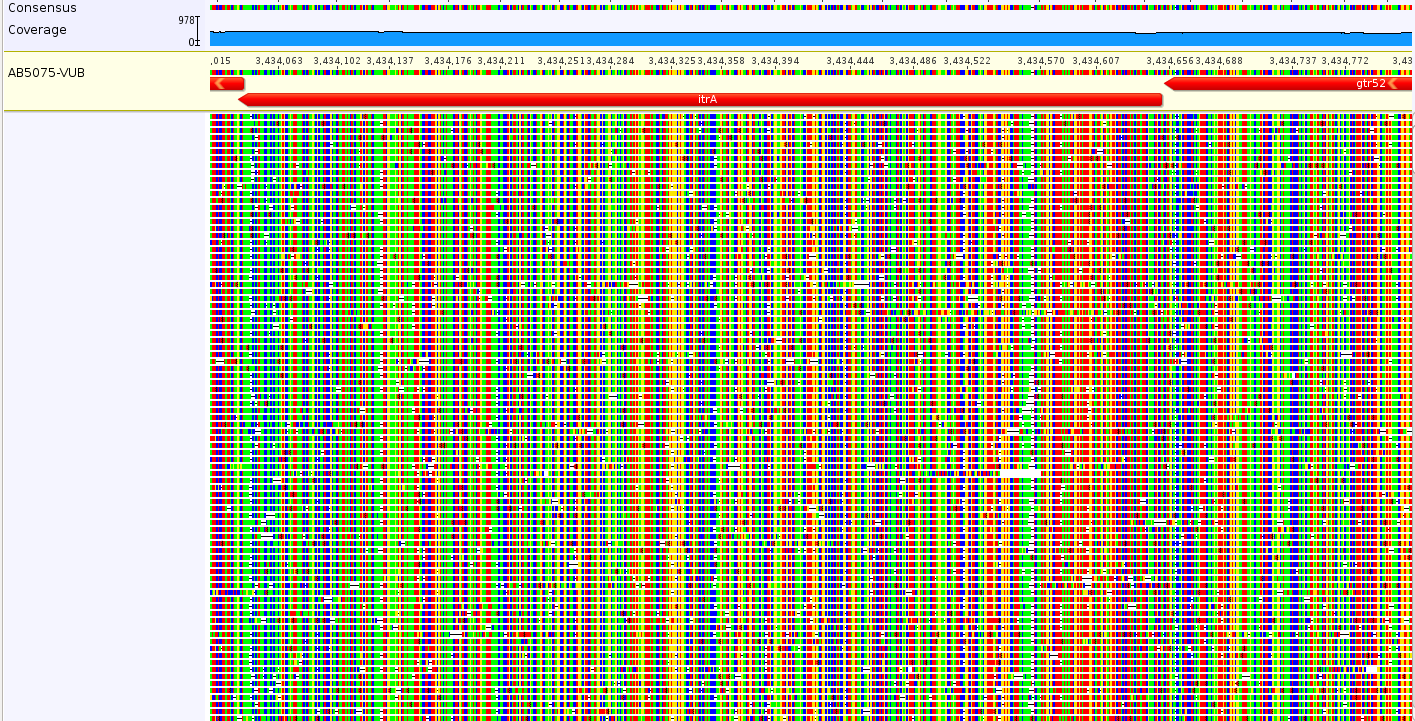


**Supplementary Fig.8 | Mapping of the short and long reads to *itrA* gene of AB5075-VUB.** This mapping is showing the coverage of the region as well as the lack of genetic variations present in this region.


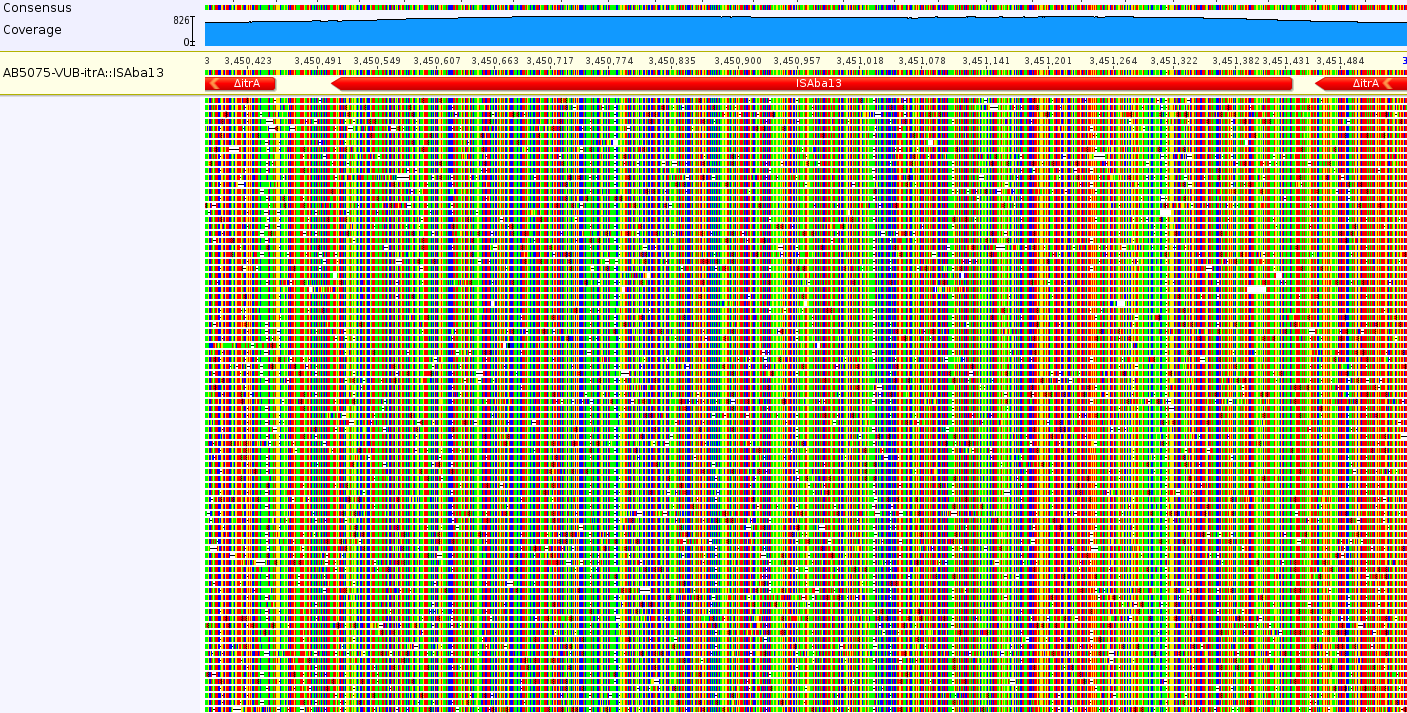


**Supplementary Fig.9 | Mapping of the short and long reads to IS*Aba13* and Δ*itrA* gene of AB5075-VUB-*itrA::ISAba13*.** This mapping is showing the coverage of the region as well as the lack of genetic variations present in this region.

**Supplementary Table. 1 | Strains and plasmids**

| **Name of the strain** | **Genotype** | **Reference** |
| --- | --- | --- |
| WT | AB5075-VUB (CP070362)  BioProject - PRJNA701627 | This study |
| *itrA::ISAba13* | AB5075-VUB-*itrA::ISAba13* (CP070358)  BioProject - PRJNA701627 | This study |
| WT Δ*itrA::sacB-aaC* | Δ*itrA::sacB-aaC* intermediate strain to generate *itrA* deletion | This study |
| *itrA::ISAba13* Δ*itrA::sacB-aaC* | Δ*itrA::sacB-aaC* intermediate strain to generate *in situ* reparation | This study |
| Δ*itrA* | Δ*itrA* | This study |
| *itrA* repaired | Replacement of *itrA::ISAba13* by the WT *itrA* to repair the locus *in situ* | This study |
| Δ*itrA/sacB-aaC* | Δ*itrA Tn7::Pst-sacB-aaC* intermediate strain to clone *itrA* at the Tn7 site | This study |
| Δ*itrA/itrA* | Δ*itrA Tn7::Pst-itrA* | This study |
| *itrA::ISAba13/sacB-aaC* | *itrA::ISAba13 Tn7::sacB-aaC* intermediate strain to clone *itrA* at the Tn7 site | This study |
| *itrA::ISAba13/itrA* | *itrA::ISAba13 Tn7::Pst-itrA* | This study |
| *itrA::ISAba13/sfGFP* | *itrA::ISAba13 Tn7::Pst-sfGFP* | This study |
| Revertant CO | *itrA::ISAba13* revertant detected on solid medium with colistin Etest strip after 6 days at 37°C | This study |
| Revertant PO-B | *itrA::ISAba13* revertant detected on solid medium with Polymyxin B Etest strip after 6 days at 37°C | This study |
| **Plasmids** |  |  |
| pASG1-1 | Pst-sfGFP for constitutive and strong expression of cloned genes and *sfGFP* cloning | ^31^ |
| pMHL2 | Apramycin resistance (*aac*) and sucrose sensitivity (*sacB*) | ^31^ |

**Supplementary Table. 2 | The differences in the genome of AB5075-VUB-*itrA::ISAba13* compared to AB5075-VUB**

| **AB5075-VUB-*itrA::ISAba13*** | **Duplications** | **Position** |  | **Insertions** | **Position** |
| --- | --- | --- | --- | --- | --- |
|  | hypothetical protein CDS | 1,538,099-1,537,901 |  | hypothetical protein CDS | 1,292,837-1,293,034 |
|  | zonula occludens toxin CDS | 1,539,558-1,538,318 |  | IS*Aba13* (disrupting *itrA*) | 3,451,437-3,450,505 |
|  | DUF2523 domain containing CDS | 1,539,835-1,539,551 |  |  |  |
|  | hypothetical protein CDS | 1,541,288-1,539,838 |  | deletions | position |
|  | phage coat protein CDS | 1,541,583-1,541,381 |  | tRNA-Met | 3,149,110-3,149,034 |
|  | hypothetical protein CDS | 1,542,442-1,542,145 |  |  |  |
|  | replication initiation factor domain-containing protein CDS | 1,543,431-1,542,445 |  |  |  |
|  | hypothetical protein CDS | 1,543,820-1,543,595 |  |  |  |
|  | hypothetical protein CDS | 1,543,931-1,544,167 |  |  |  |
| **pAB5075-VUB-itrA_3** | **duplications** | **position** |  |  |  |
|  | *intI1* | 58,334-57,321 |  |  |  |
|  | *aadB* | 58,480-59,013 |  |  |  |
|  | *cmlA* | 59,268-60,527 |  |  |  |
|  | *aadA2* | 60,620-61,411 |  |  |  |
|  | *aph(3'')-Ib* | 61,441-62,244 |  |  |  |
|  | *aph(6)-I* | 62,244-63,080 |  |  |  |

**Supplementary Table 3 | Percentage of opaque clones detected from bacterial lawns with or without Colistin Etest.**

| **%** | **∆*itrA*** | | | ***itrA::ISAba13*** | | |
| --- | --- | --- | --- | --- | --- | --- |
| **Biological replicate number** | **R1** | **R2** | **R3** | **R1** | **R2** | **R3** |
| Bacterial lawn (w/o Etest CO) | 0 | 0 | 0 | 0.13 | 0 | 0 |
|  | | | | | | |
| **Biological replicate number** | **R1** | **R2** | **R3** | **R1** | **R2** | **R3** |
| Bacterial lawn (+ Etest CO) | 0 | 0 | 0 | 1.27 | 1.62 | 0.33 |

**Supplementary Table 4 | The type of insertion sequences present in the K-locus of publicly available complete genomes and disrupted gene (n/a; not applicable)**

| **Accession number** | **Insertion sequence(s)** | **Disrupted gene(s)** |
| --- | --- | --- |
| AP023077.1 | IS*Aba10* | n/a |
| CP001921.1 | IS*Aba10* | n/a |
| CP001937.2 | IS*Aba1* | n/a |
| CP009257.1 | IS*Aba27* | n/a |
| CP010779.1 | IS*Aba1* | n/a |
| CP018677.1 | IS*Aba10* | *gtr20* |
| CP019217.1 | IS*Aba1* | *frpA* |
| CP021321.1 | IS*Aba1* | *frpA* |
| CP021326.1 | IS*Aba1* | n/a |
| CP026125.1 | IS*Aba26* | n/a |
| CP026711.1 | IS*Aba27*, IS*Aba27*, IS*Aba27* | *gtr6*, n/a, n/a |
| CP027704.1 | 𝛥IS*Acra1* | n/a |
| CP028138.1 | IS*Aba44* | *gdr* |
| CP031444.1 | IS*Aba36* | *gpi* |
| CP032743.1 | IS*Aba1* | *frpA* |
| CP033754.1 | IS*Aba22* | n/a |
| CP034242.1 | IS*Aba1* | n/a |
| CP034243.1 | IS*Aba1* | n/a |
| CP040084.1 | IS*Aba125* | *wzy* |
| CP046536.1 | IS*Aba26*, IS*Aba31*, IS*Aba14* | n/a, n/a, *pgt1* |
| CP050403.1 | IS*Aba125* | *alt1* |
| CP054302.1 | IS*Aba125*, IS*Aba125*, IS*Aba1*, IS*Aba1* | n/a, *wzy*, n/a, n/a |
| CP059386.1 | IS*Aba26* | n/a |

**Supplementary Table. 5 | List of primers used in this study**

|  | |  |
| --- | --- | --- |
| **Name** | **Sequence** | **Reference** |
| **Screening for IS*Aba13* insertion in *itrA*** | |  |
| IS13_forV2 | GTGCAAGCAGCAGATCATCG | This study |
| IS13_revV2 | AACTGCTGACGCACTAAAGG | This study |
| **Amplification of the *sacB-aaC* cassette** | |  |
| K7_for | CGACTCACTATAGGGCGAATTGGGCCGCTTTCCAGTCGGGAAACCTG | This study |
| K7_rev | CATATGCCACCGACCCGAGCAAACCCCGCCAGGGTTTTCCCAGTCACGAC | This study |
| ***itrA* deletion and repair** | |  |
| itrA_up_for | AGTAGAGGTTTTATTGTGCCTGTAC | This study |
| itrA_up_rev | GGCCCAATTCGCCCTATAGTGAGTCGAAGGACTAATGCAGTAGAAGCAATC | This study |
| itrA_down_for | GGGTTTGCTCGGGTCGGTGGCATATGATGAGTAAATTTACAGGCTCAGAAG | This study |
| itrA_down_rev | TTGACTCAAAGTCGGATGAACC | This study |
| itrA_del_for | GATTGCTTCTACTGCATTAGTCCTTATGAGTAAATTTACAGGCTCAGAA | This study |
| itrA_del_rev | CTTCTGAGCCTGTAAATTTACTCATAAGGACTAATGCAGTAGAAGCAATC | This study |
| **Cloning at the *attTn7* site** | |  |
| Tn7_up_forV2 | AGAAGGCGATATTGCCCGTCTTACTC | This study |
| Tn7_up_rev | GGCCCAATTCGCCCTATAGTGAGTCGCTGACTTCGGCTACACATCTAGAATTG | This study |
| Tn7_down_for | GGGTTTGCTCGGGTCGGTGGCATATGGTGATCTTAGGGGGTGATAATTCTG | This study |
| Tn7_down_revV2 | ATGTAGACGGGAATGGCCGGTTACG | This study |
| Tn7_ptac_for | GCAATTCTAGATGTGTAGCCGAAGTCAGgtgttgacaattaatcatcgg | This study |
| Tn7_ptac_rev | ccgatgattaattgtcaacacCTGACTTCGGCTACACATCTAGAATTGC | This study |
| Ptac_itrA_for | ctagatttaagaaggagatatacatATGCTGAAACGTTTATTAGATATTG | This study |
| Ptac_itrA_rev | CAATATCTAATAAACGTTTCAGCATatgtatatctccttcttaaatctag | This study |
| itrA_Tn7_for | GAGAAGGAAATAGAGAAAAAATGAGTGATCTTAGGGGGTGATAATTCTG | This study |
| itrA_Tn7_rev | CAGAATTATCACCCCCTAAGATCACTCATTTTTTCTCTATTTCCTTCTC | This study |
| Pst-GFP_Tn7_for | ggcatggatgagctctacaaataaGTGATCTTAGGGGGTGATAATTCTG | This study |
| Pst-GFP_Tn7_rev | CAGAATTATCACCCCCTAAGATCACttatttgtagagctcatccatgcc | This study |
